# Selective Recovery of Polyphenols from Discarded Blueberries (*Vaccinium corymbosum* L.) Using Hot Pressurized Liquid Extraction Combined with Isopropanol as an Environmentally Friendly Solvent

**DOI:** 10.3390/foods12193694

**Published:** 2023-10-08

**Authors:** Nils Leander Huamán-Castilla, Cecilia Copa-Chipana, Luis Omar Mamani-Apaza, Olivia Magaly Luque-Vilca, Clara Nely Campos-Quiróz, Franz Zirena-Vilca, María Salomé Mariotti-Celis

**Affiliations:** 1 Escuela de Ingeniería Agroindustrial, Universidad Nacional de Moquegua, Prolongación Calle Ancash s/n, Moquegua 18001, Peru; ceciliacopa1992@gmail.com (C.C.-C.); mluis1707@gmail.com (L.O.M.-A.); fzirenav@unam.edu.pe (F.Z.-V.); 2 Laboratorio de Tecnologías Sustentables para la Extracción de Compuestos de Alto Valor, Instituto de Investigación para el Desarrollo del Perú (IINDEP), Universidad Nacional de Moquegua, Moquegua 18001, Peru; 3 Escuela de Ingeniería en Industrias Alimentarias, Universidad Nacional de Juliaca, Av. Nueva Zelandia 631, Juliaca 21101, Peru; oluque@unaj.edu.pe; 4 Laboratorio de Contaminantes Orgánicos y Ambiente, Instituto de Investigación para el Desarrollo del Perú (IINDEP), Universidad Nacional de Moquegua, Moquegua 18001, Peru; claracampos23@gmail.com; 5 Escuela de Nutrición y Dietética, Facultad de Medicina, Universidad Finis Terrae, Santiago 7501015, Chile

**Keywords:** discarded blueberries, isopropanol, subcritical conditions, polyphenols, antioxidant capacity

## Abstract

The use of water–ethanol mixtures in hot pressurized liquid extraction (HPLE) to recover phenolic compounds from agro-industrial waste has been successfully investigated. However, the unresolved challenge of reducing solvent costs associated with the process hinders the scaling of this eco-friendly technology. This study evaluated the use of isopropanol as an alternative, lower-cost solvent for recovering polyphenols from discarded blueberries through the HPLE process. HPLE was carried out using water–isopropanol mixtures (0, 15 and 30%) at 70, 100, and 130 °C. The total polyphenol content (TPC), antioxidant capacity (DPPH and ORAC), glucose and fructose contents, and polyphenol profile of the extracts were determined. HPLE extracts obtained using high isopropanol concentrations (30%) and high temperatures (130 °C) presented the highest TPC (13.57 mg GAE/gdw) and antioxidant capacity (IC50: 9.97 mg/mL, ORAC: 246.47 µmol ET/gdw). Moreover, the use of 30% water–isopropanol resulted in higher yields of polyphenols and removal of reducing sugars compared to atmospheric extraction with water–acetone (60%). The polyphenolic profiles of the extracts showed that flavanols and phenolic acids were more soluble at high concentrations of isopropanol (30%). Contrarily, flavonols and stilbenes were better recovered with 15% isopropanol and pure water. Therefore, isopropanol could be a promising solvent for the selective recovery of different bioactive compounds from discarded blueberries and other agro-industrial residues.

## 1. Introduction

Peru produces ~146,000 tons of blueberries (*Vaccinium corymbosum* L.) per year, and the majority of this production (~80%) is exported [1]. However, a significant portion of blueberries is discarded during processing and packaging due to overripeness, mechanical damage, and other defects. These residues are a rich source of polyphenols, including anthocyanins, flavonols, flavanols, and phenolic acids, which have various health benefits [2,3]. For example, anthocyanins such as malvidin and cyanidin inhibit the activity of the enzyme α-glucosidase. Thus, both polyphenols are used for the treatment of diabetes [4]. Flavonols like quercetin and kaempferol are antioxidant agents which are used to treatment gastric cancer [5]. Flavanols like catechin and epicatechin have the potential to effectively mitigate excessive oxidative stress, as well as facilitating the activation of essential antioxidant components, such as glutathione peroxidases and glutathione, thereby reducing oxidative damage to the colon [6]. Thus, developing sustainable extraction methods that allow for the recovery of the polyphenols present in discarded blueberries continues to be a pending task to be resolved.

Different solvents have been used to recover phenolic compounds from different plant matrices [7]. Methanol and acetone stand out as the primary solvents employed in solid–liquid atmospheric extraction processes. Their low polarity makes them particularly well-suited for interacting with non-polar groups, such as the aromatic rings found in polyphenols, thus facilitating efficient extraction [8,9]. Thus, atmospheric extraction with aqueous methanol and acetone mixtures (30 °C for 4 h) has been applied in order to extract polyphenols from blueberry residues [10]. Nonetheless, the toxicity of these solvents renders them unsuitable for food sector applications [11]. In this sense, food-grade polyphenolic extracts have been obtained from these residues using atmospheric extraction with water–ethanol mixtures at 80 °C for periods longer than 4 h [12,13]. However, these processes require large solvent volumes and prolonged process times, which substantially raise the production costs, impeding their scaling. 

Alternative technologies, including ultrasound, microwaves, pressurized liquids, and supercritical fluids, have been developed to enhance the efficiency of polyphenol extraction when compared to traditional atmospheric methods [14]. Although alternative methods often employ shorter processing times and reduced solvent volumes, it is noteworthy that hot pressurized liquid extraction (HPLE) stands out with the most economical process costs when compared to other techniques [15,16]. The production costs for 1 g of phenolic compounds vary significantly across different extraction methods: atmospheric extraction with agitation costs USD 3.80, Soxhlet extraction costs USD 9.23, ultrasound-assisted extraction costs USD 3.70, and pressurized liquid extraction costs USD 1.32 [15,16]. HPLE is an alternative technology in which the extraction solvent is used at subcritical conditions (high pressure: 10 atm and temperatures 90–200 °C) to shorten the processing times (<20 min) [14,17]. HPLE (>120 °C), using water as a solvent, increased the yield of polyphenol extraction threefold compared to atmospheric extraction with acetone mixtures (60% acetone, 30 °C, 4 h) [18]. However, the high temperatures applied during this process generated unwanted compounds (hydroxymethyl furfural: HMF), increased the reducing sugar recovery, and hydrolyzed high-molecular-weight polyphenols [18]. 

Regarding this, the use of water–ethanol mixtures during HPLE allowed us to decrease the extraction temperature, thus avoiding the formation of unwanted compounds without reducing the polyphenol yield extraction [18,19,20,21]. However, using ethanol as an extraction solvent increases the process costs, limiting its use at an industrial level. 

Recently, the use of isopropanol during the atmospheric solid–liquid extraction of different agro-industrial residues allowed for a higher recovery of polyphenols compared to acetone, hexane, methanol, and ethanol [22,23]. Considering the significantly lower cost of isopropanol (two times lower than ethanol) [24], its use as a co-solvent during the HPLE of various vegetable matrices seems to be an attractive alternative for enhancing the extraction of polyphenols while reducing production costs. 

This research evaluated the impact of using water–isopropanol mixtures at different temperatures during HPLE on the total polyphenol content, antioxidant capacity, reducing sugars content, and phenolic profile of extracts obtained from discarded Peruvian blueberries.

## 2. Materials and Methods

### 2.1. Samples

Consorcio Agrícola Moquegua S.A.C., located in the Moquegua Region of Peru, provided 5 kg of discarded blueberries. Afterwards, the samples were frozen at −20 °C. Then, the samples were ground to a particle size of 2 mm using a grinder (MS6CA4120 ErgoMixx 800W, Bosch, München, Germany).

### 2.2. Chemical Reagents

Sigma Aldrich Chemical Co. (St. Louis, MO, USA) provided Folin–Ciocalteu reagents, sodium carbonate, fructose, glucose, DPPH (2,2-Diphenyl-1-picrylhydrazyl), AAPH (2,2′-azobis (2-methyl-propanimidamide) dihydrochloride), fluorescein, and Trolox. In addition, J.T. Baker Chemical Co. (Temixco, Mexico) supplied solvents, including methanol (≥99%) and ethanol (≥99%), while Sigma Aldrich Chemical Co. (St. Louis, MO, USA) supplied formic acid (≥98%), isopropanol (≥99%), acetonitrile (≥99%), and acetone (≥98%). Specific polyphenols, such as quercitin (≥95%), were purchased from the HWI group (Rülzheim, Germany); rutin (≥97%), kaempferol (≥97%), catechin (≥97%), and epicatechin (≥98%) were purchased from Toronto Research Chemicals Inc. (Toronto, ON, Canada); procyanidin A2 (≥90%) and procyanidin B2 (≥90%) were also purchased from Sigma Aldrich Chemical Co. (St. Louis, MO, USA); and caffeic acid (≥98%), vanillic acid (≥98%), and resveratrol (≥99%), were also purchased from TCI America (Portland, OR, USA). 

### 2.3. Hot Pressurized Liquid Extraction (HPLE)

The extraction was carried out according to the method proposed by Huaman Castilla et al. [20], with some modifications. In brief, a sample of 10 g was mixed with 10 g of neutral quartz sand to disperse the sample. Next, the mixture was placed into a 100 mL extraction cell and subjected to HPLE using an Accelerated Solvent Extraction system (ASE 150, Dionex, Thermo Fisher, San Jose, CA, USA). Polyphenols were extracted using water–isopropanol mixtures (0, 15, 30%) and high temperatures (70, 100, 130 °C) at ~10 atm. The static extraction time was 5 min, followed by rinsing with 100 mL of solvent and purging with pressurized nitrogen. The collected extracts were subjected to centrifugation at 5000 rpm for 5 min, resulting in the separation of the supernatant, which was then collected and stored in amber vials at a temperature of −20 °C before chemical analysis.

### 2.4. Total Polyphenol Content (TPC)

The TPCs of the extracts were determined according to the methodology proposed by Singleton et al. [25]. Specifically, 3.75 mL of pure water, 0.25 of the extract, 0.25 mL of Folin–Ciocalteu reagent (1:1 *v*/*v*), and 0.5 mL of sodium carbonate (10% *w*/*v*) were mixed. Then, absorbance was measured at 765 nm (Genesys 150, Thermo Fisher, San Jose, CA, USA) after a reaction time of 1 h at 20 °C. The results were expressed as mg of GAE per gram of dry weight.

### 2.5. Antioxidant Capacity by 2,2 Diphenyl 1 Picrylhydrazyl (DPPH) Analysis

The antioxidant capacity of the extracts was determined using the Brand–Williams method [26]. First, 0.1 mL of a diluted extract was mixed with 3.9 mL of 0.1 mM DPPH solution. The mixture was then protected from light for 30 min at room temperature. The reduction in the DPPH radical was measured at 517 nm using a Visible Genesys 150 UV Spectrometer (Thermo Fisher, San Jose, CA, USA). A blank was prepared using 3.9 mL of methanol and 0.1 mL of extract, while the control contained 3.9 mL of DPPH solution and 0.1 mL of methanol. Finally, the IC50 value was calculated as the concentration of antioxidant compounds required to inhibit 50% of the DPPH radical activity.

### 2.6. Antioxidant Capacity by Oxygen Radical Absorbance Capacity (ORAC) Analysis

The analysis was conducted following the methodology proposed by Chirinos et al. [27]. To generate peroxyl radicals, AAPH was utilized and Trolox was used as a standard, while fluorescein served as a fluorescence emitter. Before testing, a 48 nM fluorescein solution and 153 nM AAPH solution were diluted in a PBS buffer solution (pH 7.4). A blank sample of 25 µL of standard Trolox solution or diluted extract was combined and incubated at 37 °C for 10 min before being automatically injected into the microplate reader. Fluorescence readings were taken every minute for 50 min at 485 nm (λ: excitation) and 520 nm (emission) using a microplate reader (Synergy/HTX, Biotek Instruments Inc, Winooski, VT, USA). The final ORAC values were computed using the net area under the decay curve and expressed as µmol Trolox equivalents (ET) per gram of dry weight.

### 2.7. Quantification of Fructose and Glucose

The contents of fructose and glucose in the extracts were measured following the methodology proposed by Mariotti et al. [18]. The samples were mixed with MiliQ water at a ratio of 3:2 and centrifuged at 4025 rpm for 10 min at 4 °C. The supernatant was filtered and mixed with acetonitrile (ratio of 3:7) before being injected into an HPLC-IR system (Ultimate 3000, Dionex Thermo Scientific, Sunnyvale, CA, USA) equipped with a normal phase Li ChroCART 250-4 Purospher STAR (5 μm) column, which was maintained at 40 °C. Chromatographic separations were achieved using isocratic conditions, with an acetonitrile solution (70% *v*/*v*) at a flow rate of 1 mL/min and an injection volume of 20 μL. The results are expressed in milligrams of reducing sugar (fructose/glucose) per gram dry weight.

### 2.8. Quantification of Target Polyphenols

Specific polyphenols were quantified according to the methodology of Maldonado et al. [28] with some modifications. First, 100 µL samples were diluted with methanol and filtered through a 0.22 mm membrane. Then, 2 µL of the filtered sample was injected into an ultra-performance liquid chromatographer (Agilent 1290 II, Santa Clara, CA, USA) equipped with a diode array detector and reverse phase Poroshell C18 column (2.1 µm × 150 mm × 1.9 µm) at 30 °C. Chromatographic separation was carried out using a mobile phase consisting of A (acetonitrile and formic acid 0.1%) and B (water and formic acid 0.1%) in a gradient elution analysis programmed as follows: 95% A–5% B for 15 min, then 60% A–40% B for 18 min, and 95% A–5% B maintained for 20 min at a flow rate of 0.3 mL/min. The phenolic standards, including rutin, quercetin, caffeic acid, catechin, procyanidin B2, epicatechin, vanillic acid, procyanidin A2, resveratrol, kaempferol, were mixed and diluted to achieve a range of concentrations from 0.01 to 5.00 µg/mL. Calibration curves were constructed by correlating the peak area of each standard with its respective concentration (Table 1). Analyses were performed in triplicate, and the results are expressed in µg of the specific polyphenol per gram dry weight.

### 2.9. Statistical Analysis

A full factorial design was utilized to evaluate the impact of temperature and isopropanol concentration on the response variables. The mean of three replicates (n: 3) and the coefficient variation were used to report the results. An analysis of variance (ANOVA) and Tukey’s test were conducted for the response variables using Statgraphics Plus version 4.0, statistical software for Windows (Statpoint Technologies, Inc., Warrenton, VA, USA). 

## 3. Results and Discussion

### 3.1. Polyphenol Extraction

The increase in extraction temperature had a positive impact on the extraction of polyphenols from plant material, increasing the solvent’s kinetic energy, facilitating the rupture of the cell walls, and enhancing the solubility of polyphenols [29]. The results show that when the temperature was increased from 70 to 130 °C, the recovery of polyphenols was enhanced by 3.5, 4.2, and 5.7 times with 0 (pure water), 15% and 30% of isopropanol, respectively (Table 2). 

Although there are no reports on the effect of isopropanol under subcritical conditions, previous studies have investigated its impact under atmospheric conditions (1 atm). For example, Bánvölgyi et al. [30] have demonstrated that an increase from 30 to 60 °C with 50% isopropanol improved the total polyphenol content by ~9.2 times compared to pure water under the same conditions. Subra-Paternault et al. [31] reported that the use of 60% isopropanol allowed for the recovery of 77% more total polyphenol content compared to use of 86% ethanol at 60 °C. Isopropanol presents a lower dielectric constant (ε: 19.92) compared to pure water (ε: 80) and ethanol (ε: 24.3) [32,33]. Thus, the presence of isopropanol in the solvent reduces its polarity, improving the solubilization of polyphenols. Additionally, isopropanol presents two functional groups (polar and nonpolar), where the hydroxyl group (polar) and isopropyl group (nonpolar) can interact with the hydroxyl groups and aromatic rings of the polyphenols, respectively. Consequently, the solubility of polyphenols can be improved [31,34].

### 3.2. Antioxidant Capacity

Polyphenols’ ability to inhibit specific radicals can be evaluated using the DPPH and ORAC methods. The DPPH method measures polyphenols’ capacity to neutralize DPPH, a free radical, which is distinct from other biological reactive species (peroxyl radicals), while the ORAC method assesses polyphenols’ capacity to neutralize peroxyl radicals [19]. 

Table 2 shows that an increase in temperature and a higher concentration of isopropanol improved the antioxidant capacity. For example, when temperature was increased from 70 to 130 °C, the antioxidant capacity increased by 64 and 126 times with pure water and 30% of isopropanol, respectively (Table 2). 

Perović et al. [35] reported that under atmospheric conditions, an increase from 30 to 45 °C combined with 50% water–isopropanol mixture improved the antioxidant capacity of the extracts by approximately 2.9 times compared to using pure water. The addition of isopropanol likely promoted more interactions between the functional groups of polyphenols and the solvent, especially with compounds of intermediate polarities, such as anthocyanins and flavonol monomers [36,37]. 

In general, a smaller IC_50_ value signifies a greater ability to inhibit DPPH radical activity [18,21]. For DPPH analysis, the blueberry residue extracts presented a lower IC_50_ value when HPLE was carried out using higher isopropanol concentrations and temperatures (Table 2). The best condition was established with 30% isopropanol at 130 °C (IC_50_: 9.97 mg/mL); these results indicate that only 9.97 mg of the extract is required to reduce the activity of the DPPH radical by 50%.

### 3.3. Reducing Sugar Content

**Figure 1:**
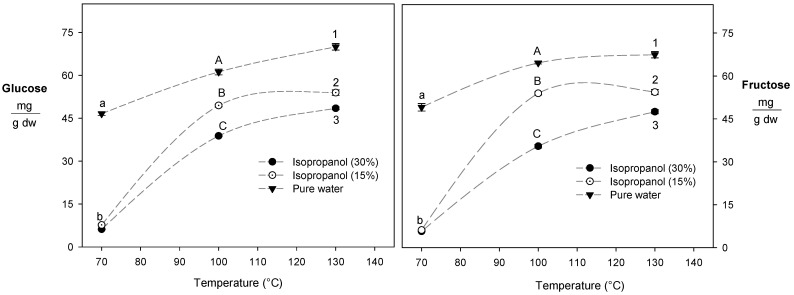
Effect of isopropanol content on the recovery of reducing sugars. Different letters indicate statistically significant differences (*p* < 0.05). Lowercase letters, capital letters, and numbers indicate differences for 70, 100, and 130 °C, respectively.

An increase in the extraction temperature during the HPLE of blueberry residues enhanced the recovery of reducing sugars. However, higher isopropanol concentrations decreased (30%) the glucose and fructose content of the extracts (Figure 1). Similarly, during the HPLE of grape pomace, the presence of reducing sugars in the extracts was reduced by up to 13% if the concentration of ethanol exceeded 15% [18]. The addition of intermediate polarity solvents such as ethanol and glycerol reduced the solvent’s polarity, which can hinder interactions between the water molecules and reduce sugars [20]. In this sense, isopropanol has a lower dielectric constant (ε: 19.92) compared to ethanol (ε: 24.3) and glycerol (ε: 42.5) at 30 °C [33], which could explained its lower selectivity for glucose and fructose.

### 3.4. Impact of the Use of Isopropanol to Recover Specific Polyphenols

#### 3.4.1. Flavonols

Temperature and solvent composition affected the content of flavonols, whose values varied from 9.92 µg/gdw to 333.82 µg/gdw. Additionally, when the temperature was increased from 70 to 100 °C using a 30% water–isopropanol mixture as the extraction solvent, the flavonol content of blueberry residue extracts increased by 2.2 times. However, when the temperature changed from 100 °C to 130 °C, the flavonol content decreased by approximately 1.9 times (Table 3). A similar trend was observed when grape skin was submitted to HPLE with water. The recovery of anthocyanins and flavanols resulted in a 24% decrease when the extraction temperature exceeded 120 °C [38].

The best HPLE conditions for the selective recovery of flavonols were 100 °C and 30% of isopropanol, in which the extracts contained the highest proportion (>50%) of quercetin (Table 3), the most abundant polyphenol in blueberries [39].

#### 3.4.2. Flavanols

The highest yield of flavanol extraction (53.51 μg/gdw) was achieved when HPLE was carried out at the highest temperature (130 °C) and isopropanol concentration (30%). Under these conditions, the flavanol content of blueberry residue extract increased by ~16 times compared to that obtained with pure water under same conditions (Table 3). Interestingly, the extracts contained epicatechin, catechin, procyanidin A2, and procyanidin B2, with epicatechin being the major polyphenol at 22.82 μg/gdw (Table 3). 

Although there is no information regarding the efficacy of water–isopropanol mixtures for flavanol recovery, recent studies have highlighted the utility of co-solvents like ethanol and glycerol for reducing the solvent’s polarity and improving the extractability of these compounds [20,40]. 

#### 3.4.3. Phenolic Acids

Under subcritical conditions, the highest recovery of phenolic acids was achieved at the highest temperature (130 °C), combined with 30% isopropanol (Table 3). When the temperature increased from 70 to 130 °C with 30% isopropanol, the recovery of these compounds increased by 150% compared to pure water (Table 3). Ju and Howard [37] reported, under subcritical conditions in red grape skin samples, that an increase from 100 to 140 °C improves phenolic acid recovery by 28%, using pure water as extraction solvent. The highest recovery of caffeic acid (1.71 µg/gss) and vanillic acid (9.49 µg/gss) was achieved under these conditions (130 °C, 30% of isoproponal). Previous studies on blueberries have also highlighted the significance of phenolic acids, particularly vanillic and caffeic acid, as major polyphenols [41,42,43].

#### 3.4.4. Stilbenes

In contrast to other specific families, the extraction of stilbenes exhibited a different pattern. Our study found that the maximum recovery of resveratrol (4.28 μg/gdw) was achieved using the highest temperature (130 °C) combined with pure water. 

Previous studies have reported that the use of high ethanol concentrations (>32.5%) negatively impacts the recuperation of stilbenes [20]. It is likely that the presence of a single hydroxyl group in ethanol and isopropanol molecules reduces their ability to solubilize these compounds. Consequently, a lower proportion of these compounds is recovered.

### 3.5. Impact of the Use of Isopropanol versus Ethanol in HPLE and Conventional Extraction with Acetone

**Figure 2:**
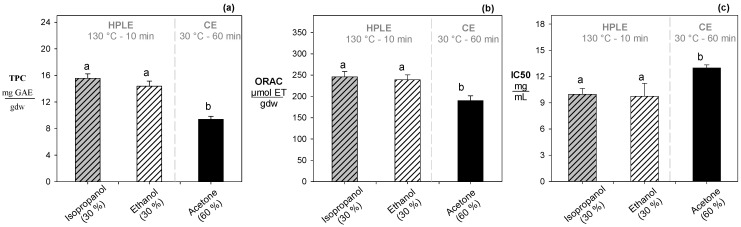
Effect of isopropanol content on the recovery of reducing sugars. (**a**) analysis for total polyphenol content. (**b**) analysis for antioxidant capacity by ORAC. (**c**) analysis for antioxidant capacity by IC50. Different letters indicate statistically significant differences (*p* < 0.05).

Under subcritical conditions at 130 °C, the use of co-solvents such as isopropanol (30%) and ethanol (30%) did not yield significant differences in polyphenol content or antioxidant capacity in terms of ORAC and IC_50_ (Figure 2). Isopropanol presents a lower dielectric constant (ε: 19.92) when compared to ethanol (ε: 24.06) [44]. Consequently, the use of isopropanol should have a greater affinity for compounds of intermediate polarity (polyphenols). However, for a correct analysis, it is imperative to take into account intermolecular interactions, including hydrogen bonds and interactions among non-polar groups. These intermolecular interactions can be evaluated using the solvatochromic parameters of both solvents. In this sense, ethanol and isopropanol exhibit a similar capacity for form hydrogen bonds (α), with 0.83 for ethanol and 0.78 for isopropanol, which elucidates the observed behavior of both solvents.

On the other hand, the use of isopropanol (30%) enhanced the total polyphenol content and antioxidant capacity (ORAC) compared to the use of acetone (60%) by ~23% and ~15%. On the contrary, the DPPH values were reduced by 19% (Figure 2). Although both solvent isopropanol (ε: 19.92) and acetone (ε: 20.70) were similar [44], isopropanol exhibited higher acidity (α: 0.78) compared to acetone (α: 0.08) [45]. The acidity is a solvatochromic parameter that measure the solvent’s ability to form hydrogen bonds with other functional groups [45,46], which may explain the superior ability of isopropanol to recover intermediate polarity compounds such as polyphenols.

## 4. Conclusions

Under subcritical conditions, the use of high concentrations of isopropanol (30%) combined with elevated temperatures (130 °C) facilitated the extraction of compounds with high concentrations of polyphenols with important antioxidant properties from discarded blueberries. Furthermore, heightened levels of isopropanol contributed to a decrease in the concentration of reducing sugars, minimizing the presence of these undesired compounds. Moreover, the solvent composition allowed for the selective recovery of distinct polyphenolic families. Specifically, a heightened concentration of isopropanol facilitated the retrieval of flavonols, flavones, and phenolic acids. Conversely, the utilization of pure water resulted in the optimal recovery of stilbenes. No significant differences were observed between the use of isopropanol and ethanol during HPLE in the recovery of antioxidant compounds. Finally, the utilization of isopropanol is emerging as a novel and cost-effective avenue for the development of eco-friendly and economically viable extraction processes.

## Figures and Tables

**Table 1:** Analytical characteristics of the high-performance liquid chromatography method coupled to a diode array detector for the quantification of specific polyphenols.

Specific Polyphenol	Wavelength (nm)	Regression Equation	R^2^
Rutin	270	Y = 6.6151462X + 1.7802541	0.99922
Quercitin	270	Y = 24.4618691X + 2.2829876	0.99971
Caffeic acid	270	Y = 149.119813X + 0.9753017	0.99994
Catechin	280	Y = 25.251136X − 0.5309875	0.99994
Procyanidin B2	280	Y = 41.3596684X − 0.4846145	0.99974
Epicatechin	280	Y = 43.3950296X − 2.1554659	0.99985
Vanillic acid	280	Y = 141.991849X − 5.4568242	0.99980
Procyanidin A2	280	Y = 59.2803924X − 0.7963507	0.99990
Resveratrol	324	Y = 78.8100873X − 31.357898	0.99978
Kaempferol	373	Y = 38.0226353X − 1.5363721	0.99971

**Table 2:** Chemical characterization of the extracts.

Conditions	70 °C			100 °C			130 °C		
Isopropanol (%)	TPC	IC_50_	ORAC	TPC	IC_50_	ORAC	TPC	IC_50_	ORAC
	Mean CV	Mean CV	Mean CV	Mean CV	Mean CV	Mean CV	Mean CV	Mean CV	Mean CV
**0**	2.32 ^A,a^	20.44 ^C,a^	90.85 ^A,a^	3.61 ^B,a^	16.49 ^B,c^	121.85 ^B,a^	8.13 ^C,a^	13.61 ^A,b^	149.90 ^C,a^
	0.09	0.02	0.03	0.04	0.03	0.04	0.08	0.03	0.05
**15**	2.66 ^A,b^	19.22 ^C,a^	97.59 ^A,b^	6.30 ^B,b^	14.24 ^B,b^	148.59 ^B,b^	11.26 ^C,b^	12.23 ^A,b^	169.33 ^C,b^
	0.07	0.03	0.04	0.08	0.03	0.05	0.04	0.03	0.03
**30**	2.71 ^A,b^	17.99 ^C,b^	108.52 ^A,c^	12.18 ^B,c^	11.43 ^B,a^	211.47 ^B,c^	15.57 ^C,c^	9.97 ^A,a^	246.29 ^C,c^
	0.09	0.03	0.03	0.05	0.04	0.05	0.02	0.04	0.06

TPC: Total polyphenol content is expressed as mg of gallic acid equivalent per gram of dry weight. IC_50_ is expressed as mg of extract to inhibit 50% of the DPPH radical solution (mL). ORAC was expressed as µmol Trolox equivalent per gram of dry weight. The results are expressed as the mean and CV (coefficient variation). Different letters indicate statistically significant differences (*p* < 0.05). Lowercase letters indicate differences between solvent concentrations. Capital letters indicate differences between processing temperatures.

**Table 3:** Polyphenolic profile of the obtained extracts.

Temperature	70 °C			100 °C			130 °C		
Isopropanol	0%	15%	30%	0%	15%	30%	0%	15%	30%
**Flavanols (µg/gdw)**	Mean CV	Mean CV	Mean CV	Mean CV	Mean CV	Mean CV	Mean CV	Mean CV	Mean CV
Quercetin	2.03	77.09	76.96	10.63	88.93	171.12	24.98	121.05	78.1
	0.02	0.03	0.02	0.08	0.06	0.11	0.09	0.05	0.02
Rutin	ND	14.43	71.06	4.76	22.08	161.19	ND	64.45	93.37
		0.10	0.01	0.10	0.10	0.10		0.02	0.03
Kaempferol	0.91	0.98	0.93	1.25	1.09	1.51	1.46	1.73	1.64
	0.01	0.05	0.05	0.10	0.01	0.02	0.05	0.05	0.11
**∑:**	**2.92**	**92.5**	**148.92**	**16.64**	**112.11**	**333.82**	**26.44**	**187.23**	**173.11**
**Flavanols (µg/gdw)**									
Catechin	0.53	0.66	10.46	0.55	1.08	17.36	1.00	1.29	18.67
	0.10	0.09	0.08	0.01	0.06	0.03	0.01	0.01	0.02
Epicatechin	ND	1.41	11.69	ND	1.47	18.48	ND	3.40	22.82
		0.01	0.05		0.09	0.04		0.03	0.05
Procyanidin A2	0.45	0.71	0.79	0.71	1.50	2.55	0.74	2.15	1.10
	0.07	0.04	0.01	0.10	0.01	0.08	0.04	0.02	0.11
Procyanidin B2	ND	1.87	3.20	1.28	2.09	4.11	1.46	3.09	10.91
		0.04	0.11	0.07	0.00	0.11	0.03	0.04	0.02
**∑:**	**0.99**	**4.65**	**26.13**	**2.55**	**6.14**	**42.49**	**3.20**	**9.92**	**53.51**
**Phenolic acids** **(ug/gdw)**									
Caffeic	ND	0.02	1.26	ND	0.05	1.43	0.01	0.30	1.71
		0.07	0.05		0.02	0.07	0.09	0.09	0.06
Vanillic	0.96	1.01	1.30	2.02	1.83	3.50	7.39	8.59	9.49
	0.01	0.06	0.00	0.09	0.03	0.10	0.08	0.01	0.03
**∑:**	**0.96**	**1.03**	**2.56**	**2.02**	**1.88**	**4.93**	**7.40**	**8.89**	**11.20**
**Stilbens (µg/gdw)**									
Resveratrol	7.49	7.98	ND	8.15	8.01	ND	9.08	8.04	ND
	0.00	0.00		0.00	0.00		0.00	0.00	

Content is expressed as μg of specific polyphenol per gram of dry weight. ND: Not detected.

## Data Availability

Data is contained within the article.

## References

[1] INEI Informe Técnico de Producción Nacional-Diciembre 2019 [Report]. https://www.inei.gob.pe/media/MenuRecursivo/boletines/02-informe-tecnico-n02_produccion-nacional-dic-2019.pdf/.

[2] Li D., Li B., Ma Y., Sun X., Lin Y., Meng X. (2017). Polyphenols, Anthocyanins, and Flavonoids Contents and the Antioxidant Capacity of Various Cultivars of Highbush and Half-High Blueberries. J. Food Compos. Anal..

[3] Wang S.Y., Chen C.T., Sciarappa W., Wang C.Y., Camp M.J. (2008). Fruit Quality, Antioxidant Capacity, and Flavonoid Content of Organically and Conventionally Grown Blueberries. J. Agric. Food Chem..

[4] He K., Li X., Chen X., Ye X., Huang J., Jin Y., Li P., Deng Y., Jin Q., Shi Q. (2011). Evaluation of Antidiabetic Potential of Selected Traditional Chinese Medicines in STZ-Induced Diabetic Mice. J. Ethnopharmacol..

[5] Mahmud A.R., Ema T.I., Siddiquee M.F.R., Shahriar A., Ahmed H., Mosfeq-Ul-Hasan M., Rahman N., Islam R., Uddin M.R., Mizan M.F.R. (2023). Natural Flavonols: Actions, Mechanisms, and Potential Therapeutic Utility for Various Diseases. Beni-Suef Univ. J. Basic Appl. Sci..

[6] Fan F.Y., Sang L.X., Jiang M., McPhee D.J. (2017). Catechins and Their Therapeutic Benefits to Inflammatory Bowel Disease. Molecules.

[7] Cvjetko Bubalo M., Vidović S., Radojčić Redovniković I., Jokić S. (2015). Green Solvents for Green Technologies. J. Chem. Technol. Biotechnol..

[8] Brglez Mojzer E., Knez Hrnčič M., Škerget M., Knez Ž., Bren U. (2016). Polyphenols: Extraction Methods, Antioxidative Action, Bioavailability and Anticarcinogenic Effects. Molecules.

[9] Sulaiman S.F., Sajak A.A.B., Ooi K.L., Supriatno, Seow E.M. (2011). Effect of Solvents in Extracting Polyphenols and Antioxidants of Selected Raw Vegetables. J. Food Compos. Anal..

[10] Nile S.H., Park S.W. (2014). Edible Berries: Bioactive Components and Their Effect on Human Health. Nutrition.

[11] Joshi D.R., Adhikari N. (2019). An Overview on Common Organic Solvents and Their Toxicity. J. Pharm. Res. Int..

[12] Drosou C., Kyriakopoulou K., Bimpilas A., Tsimogiannis D., Krokida M. (2015). A Comparative Study on Different Extraction Techniques to Recover Red Grape Pomace Polyphenols from Vinification Byproducts. Ind. Crops Prod..

[13] Aaby K., Grimmer S., Holtung L. (2013). Extraction of Phenolic Compounds from Bilberry (*Vaccinium myrtillus* L.) Press Residue: Effects on Phenolic Composition and Cell Proliferation. LWT—Food Sci. Technol..

[14] Ameer K., Shahbaz H.M., Kwon J.H. (2017). Green Extraction Methods for Polyphenols from Plant Matrices and Their Byproducts: A Review. Compr. Rev. Food Sci. Food Saf..

[15] Santos D.T., Veggi P.C., Meireles M.A.A. (2010). Extraction of Antioxidant Compounds from Jabuticaba (*Myrciaria Cauliflora*) Skins: Yield, Composition and Economical Evaluation. J. Food Eng..

[16] Santos D.T., Veggi P.C., Meireles M.A.A. (2012). Optimization and Economic Evaluation of Pressurized Liquid Extraction of Phenolic Compounds from Jabuticaba Skins. J. Food Eng..

[17] Plaza M., Turner C. (2015). Trends in Analytical Chemistry Pressurized Hot Water Extraction of Bioactives. Trends Anal. Chem..

[18] Mariotti-Celis M.S., Martínez-Cifuentes M., Huamán-Castilla N., Pedreschi F., Iglesias-Rebolledo N., Pérez-Correa J.R. (2018). Impact of an Integrated Process of Hot Pressurised Liquid Extraction–Macroporous Resin Purification over the Polyphenols, Hydroxymethylfurfural and Reducing Sugars Content of *Vitis vinifera* ‘Carménère’ Pomace Extracts. Int. J. Food Sci. Technol..

[19] Allcca-Alca E.E., León-Calvo N.C., Luque-Vilca O.M., Martínez-Cifuentes M., Pérez-Correa J.R., Mariotti-Celis M.S., Huamán-Castilla N.L. (2021). Hot Pressurized Liquid Extraction of Polyphenols from the Skin and Seeds of *Vitis vinifera* L. cv. Negra Criolla Pomace a Peruvian Native Pisco Industry Waste. Agronomy.

[20] Huaman-Castilla N.L., Martinez M., Camilo C., Pedreschi F., Mariotti-Celis M.S., Perez-Correa J.R. (2019). The Impact of Temperature and Ethanol Concentration on the Global Recovery of Specific Polyphenols in an Integrated HPLE/RP Process on Carm é n è Re Pomace Extracts. Molecules.

[21] Mariotti-Celis M., Martínez-Cifuentes M., Huamán-Castilla N., Vargas-González M., Pedreschi F., Pérez-Correa J. (2017). The Antioxidant and Safety Properties of Spent Coffee Ground Extracts Impacted by the Combined Hot Pressurized Liquid Extraction–Resin Purification Process. Molecules.

[22] Jayakumar C., Devi V.M., Sridar R. (2020). A Study on the Extraction of Bioactive Compounds from *Capparis zeylanica*. AIP Conf. Proc..

[23] Vimercati W.C., Araújo C.d.S., Macedo L.L., Pimenta C.J. (2022). Optimal Extraction Condition for the Recovery of Bioactive Compounds and Antioxidants from Coffee Silverskin. J. Food Process Eng..

[24] Merck: Precio Solventes Organicos (Isopropanol). https://www.sigmaaldrich.com/PE/es/search/2-propanol?focus=products&page=1&perpage=30&sort=relevance&term=2%20propanol&type=product/.

[25] Singleton V.L., Rossi J.A. (1965). Colorimetry of Total Phenolics with Phosphomolybdic-Phosphotungstic Acid Reagents. Am. J. Enol. Vitic..

[26] Brand-Williams, Cuvelier M.E., Berset C. (1995). Use of a Free Radical Method to Evaluate Antioxidant Activity. Food Sci. Technol..

[27] Chambia F., Chirinosa R., Pedreschic R., Betalleluz-Pallardela I., Debasteb F., Campos D. (2013). Antioxidant Potential of Hydrolyzed Polyphenolic Extracts from Tara (*Caesalpinia spinosa*) Pods. Ind. Crops Prod..

[28] Maldonado I., Vega Quispe A.P., Merma Chacca D., Zirena Vilca F. (2022). Optimization of the Elimination of Antibiotics by Lemna Gibba and Azolla Filiculoides Using Response Surface Methodology (RSM). Front. Environ. Sci..

[29] Vergara-Salinas J.R., Bulnes P., Zúñiga M.C., Pérez-Jiménez J., Torres J.L., Mateos-Martín M.L., Agosin E., Pérez-Correa J.R. (2013). Effect of Pressurized Hot Water Extraction on Antioxidants from Grape Pomace before and after Enological Fermentation. J. Agric. Food Chem..

[30] Bánvölgyi S., Dusza E., Namukwambi F.K., Kiss I., Stefanovits-Bányai É., Vatai G. (2020). Optimization of Extraction of Phenolic Compounds from Tokaji Aszú Marc Using Response Surface Methodology. Prog. Agric. Eng. Sci..

[31] Subra-Paternault P., Garcia-Mendoza M.D.P., Savoire R., Harscoat-Schiavo C. (2022). Impact of Hydro-Alcoholic Solvents on the Oil and Phenolics Extraction from Walnut (*Juglans regia* L.) Press-Cake and the Self-Emulsification of Extracts. Foods.

[32] Stranathan J.D. (1937). The Dielectric Constant of Isopropyl Alcohol Vapor. J. Chem. Phys..

[33] Åkerlöf G. (1932). Dielectric Constants of Some Organic Solvent-Water Mixtures at Various Temperatures. J. Am. Chem. Soc..

[34] Zhang H., Birch J., Yang H., Xie C., Kong L., Dias G., Bekhit A.E.D. (2018). Effect of Solvents on Polyphenol Recovery and Antioxidant Activity of Isolates of Asparagus Officinalis Roots from Chinese and New Zealand Cultivars. Int. J. Food Sci. Technol..

[35] Bánvölgyi1 S., Namukwambi1 F.K., István K., Stefanovits-Bányai E., Vatai1 G. Extraction of antioxidant and polyphenol compounds from Tokaji aszú marc with iso-propanol–water solvent. Proceedings of the 23rd International Symposium on Analytical and Environmental Problems.

[36] Smith M.A.L., Marley K.A., Seigler D., Singletary K.W., Meline B. (2000). Bioactive Properties of Wild Blueberry Fruits. J. Food Sci..

[37] Wu X., Beecher G.R., Holden J.M., Haytowitz D.B., Gebhardt S.E., Prior R.L. (2006). Concentrations of Anthocyanins in Common Foods in the United States and Estimation of Normal Consumption. J. Agric. Food Chem..

[38] Ju Z.Y., Howard L.R. (2003). Effects of Solvent and Temperature on Pressurized Liquid Extraction of Anthocyanins and Total Phenolics from Dried Red Grape Skin. J. Agric. Food Chem..

[39] Wang Y., Fong S.K., Singh A.P., Vorsa N., Johnson-Cicalese J. (2019). Variation of Anthocyanins, Proanthocyanidins, Flavonols, and Organic Acids in Cultivated and Wild Diploid Blueberry Species. HortScience.

[40] Huamán-Castilla N.L., Mariotti-Celis M.S., Martínez-Cifuentes M., Pérez-Correa J.R. (2020). Glycerol as Alternative Co-Solvent for Water Extraction of Polyphenols from Carménère Pomace: Hot Pressurized Liquid Extraction and Computational Chemistry Calculations. Biomolecules.

[41] Cervantes Ceja M.L. (2009). “Potencial Nutracéutico de Cultivos de Arándano (Vaccinum sp.) Seleccionados En México”, Universidad Autónoma de Querétaro. https://cdn.blueberriesconsulting.com/2015/09/pdf_310.pdf/.

[42] Brambilla A., Lo Scalzo R., Bertolo G., Torreggiani D. (2008). Steam-Blanched Highbush Blueberry (*Vaccinium corymbosum* L.) Juice: Phenolic Profile and Antioxidant Capacity in Relation to Cultivar Selection. J. Agric. Food Chem..

[43] Al Hasani S., Al-attabi Z., Waly M. (2023). Polyphenol and Flavonoid Stability of Wild Blueberry (Sideroxylon mascatense) during Air- and Freeze-Drying and Storage Stability as a Function of Temperature. Foods.

[44] Méndez-Bermúdez J.G., Dominguez H., Pusztai L., Guba S., Horváth B., Szalai I. (2016). Composition and Temperature Dependence of the Dielectric Constant of 1-Propanol/Water Mixtures: Experiment and Molecular Dynamics Simulations. J. Mol. Liq..

[45] Jessop P.G., Jessop D.A., Fu D., Phan L. (2012). Solvatochromic Parameters for Solvents of Interest in Green Chemistry. Green Chem..

[46] Jessop P.G. (2011). Searching for Green Solvents. Green Chem..

